# A new species of the genus *Falcileptoneta* Komatsu, 1970 (Araneae, Leptonetidae) from Jilin, China

**DOI:** 10.3897/BDJ.13.e143433

**Published:** 2025-01-03

**Authors:** Hao Yu, Yuanqian Xing, Yejie Lin

**Affiliations:** 1 The State Key Laboratory of Southwest Karst Mountain Biodiversity Conservation of Forestry Administration, School of life sciences, Guizhou Normal University, Guiyang, China, Guiyang, China The State Key Laboratory of Southwest Karst Mountain Biodiversity Conservation of Forestry Administration, School of life sciences, Guizhou Normal University, Guiyang, China Guiyang China; 2 Imperial College London, London, United Kingdom Imperial College London London United Kingdom

**Keywords:** type, morphology, diagnosis, troglobitic spider, taxonomy

## Abstract

**Background:**

*Falcileptoneta* Komatsu, 1970 comprises 68 species, distributed in Japan (28), Korea (31) and southeast of China (9). This genus has not been recorded in the north-eastern part of China.

**New information:**

A new species: *Falcileptonetataoqii* sp. nov. (♂♀) are reported from Jilin Province, China. This is also the first record of Leptonetidae Simon, 1890 in north-eastern China. Photos and morphological descriptions of the new species are presented; the type specimens of the new species are deposited in the Institute of Zoology, Chinese Academy of Sciences (IZCAS), Beijing.

## Introduction

The spider genus *Falcileptoneta* Komatsu, 1970 comprises 68 species and is a member of the family Leptonetidae Simon, 1890. They are commonly found in leaf litter and caves, where they construct sheet webs. The genus can be distinguished by the male femur lacking robust spines, tibia bearing 1–3 robust spines and tarsus undivided, apically blunt without a tapering process (Oh and Lee 2023).

At present, 68 species of *Falcileptoneta* are distributed in Japan (28), South Korea (31) and southeast of China (9) (World Spider Catalog 2024). Most species are endemic, thereby rendering them particularly well suited to the study of biodiversity (Oh et al. 2022). In China, except for *F.shuanglong* Wang & Li, 2020, the remaining eight species formerly belonged to the genus *Leptoneta* Simon, 1872 (Wang et al. 2020). Only three are described by both sexes. Consequently, descriptions of the unknown sex will be of great academic importance in the future.

**Figure 1.**
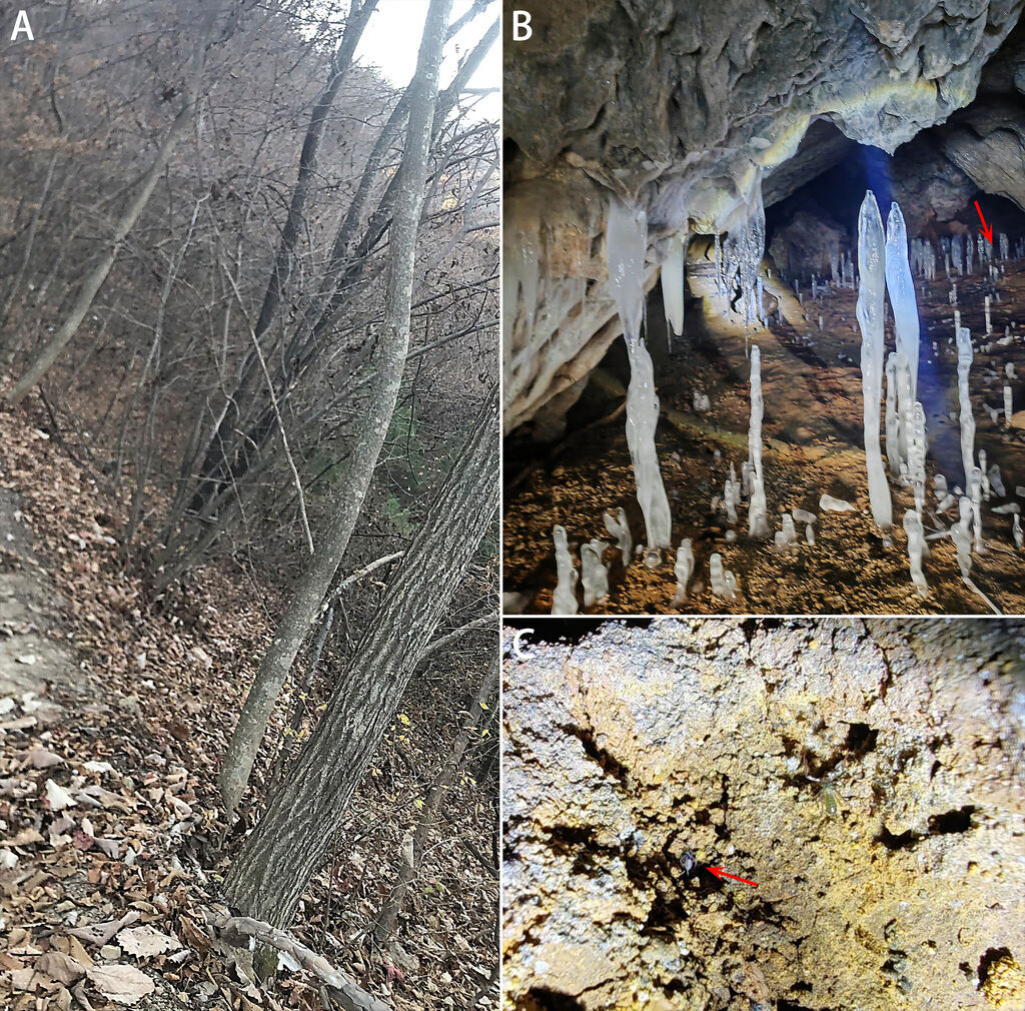
*Falcileptonetataoqii* sp. nov., habitat. **A** Environment outside the cave; **B** Interior of the cave (red arrow shows where the spider was found); **C** *F.taoqii* sp. nov. (red arrow) and *Bisetocreagris* sp. (Neobisidae). Photos by Taoqi Wang.

Given the distribution site, it might be expected that this genus of spiders would be more widely distributed in China. However, the difficulty of collecting specimens, particularly given their cryptic nature, has meant that they have not been recorded in the north-eastern part of China. In South Korea, nine species have been identified in caves. Jilin is characterised by extensive karst landforms, leading us to believe that more species of Leptonetidae will be discovered in the future. In this paper, we report a new *Falcileptoneta* species from Jilin, China (Fig. 1). This is also the first record of Leptonetidae in north-eastern China.

## Materials and methods

All specimens were preserved in 80% ethanol. The spermathecae were cleared in trypsin enzyme solution to dissolve non-chitinous tissues. Specimens were examined under a LEICA M205C stereomicroscope. Photomicrographs were taken with an Olympus C7070 zoom digital camera (7.1 megapixels). Photos were stacked with Helicon Focus (Version 7.6.1) or Zerene Stacker (Version 1.04) and processed in Adobe Photoshop CC2022.

All measurements are in millimetres and obtained with an Olympus SZX16 stereomicroscope with a Zongyuan CCD industrial camera. Measurements method follows Seo (2015). All measurements of body lengths do not include the chelicerae. Eye sizes are measured as the maximum diameter from either the dorsal or frontal view. Leg measurements are given as follows: total length (femur, patella, tibia, metatarsus, tarsus), the terminology used in the text and figures following Xu et al. (2019) and Oh and Lee (2023).

A total of 610 bases of cytochrome oxidase I were sequenced by using the following primers: LCOI1490 (5’-GGTCAACAAATCATAAAGATATTG-3’) and HCOI2198 (5’-TAAACTTCAGGGTGACCAAAAAAT-3’). This PCR profile consisted of an initial denaturing step at 95°C for 5 min, 40 amplification cycles [95°C for 30 s, 45°C or optimal annealing temperature (Tm°C) for 45 s, 72°C for 70 s], followed by a final extension step at 72°C for 10 min.

Abbreviations: **AER**, anterior eye row; **ALE**, anterior lateral eye; **AME**, anterior median eye; **At**, atrium; **C**, conductor; **E**, embolus; **MS**, median sclerite; **PER**, posterior eye row; **PL**, prolateral lobe; **PME**, posterior median eye; **PP**, pore plate **PS**, prolateral sclerite; **RTS**, retrolateral tibial spine; **S**, spermathecae; **SS**, spermathecae stalk.

Types from the current study are deposited in the Institute of Zoology, Chinese Academy of Sciences in Beijing (**IZCAS**).

## Taxon treatments

### Falcileptoneta taoqii

Yu & Lin sp. nov.

528910B9-3068-5312-8B3A-D2EDD84F3EFB

A0307ED8-287F-478C-955C-C12066C78717

#### Materials

- **Type status:** Holotype. **Occurrence:** recordedBy: Taoqi Wang; individualCount: 1; sex: male; lifeStage: adult; occurrenceID: 8631AECC-8D90-5A9F-8C0C-00FB228905AD; **Taxon:** scientificName: *Falcileptonetataoqii*; **Location:** country: China; stateProvince: Jilin; municipality: Tonghua; locality: Ji'an City, Dalu Town, Gaodi Village, Group 8, Feng Cave; verbatimElevation: 310.8 m; verbatimCoordinates: 125.8232°E, 47.0471°N; **Identification:** identifiedBy: Yejie Lin; dateIdentified: 2024; **Event:** year: 2024; month: 1; day: 24; habitat: Cave; **Record Level:** institutionCode: IZCAS-Ar45515
- **Type status:** Paratype. **Occurrence:** recordedBy: Taoqi Wang; individualCount: 1; sex: female; lifeStage: adult; occurrenceID: DDE0855A-075F-52B1-926E-CF494F9EEFF4; **Taxon:** scientificName: *Falcileptonetataoqii*; **Location:** country: China; stateProvince: Jilin; municipality: Tonghua; locality: Ji'an City, Dalu Town, Gaodi Village, Group 8, Feng Cave; verbatimElevation: 310.8 m; verbatimCoordinates: 125.8232°E, 47.0471°N; **Identification:** identifiedBy: Yejie Lin; dateIdentified: 2024; **Event:** year: 2024; month: 1; day: 24; habitat: Cave; **Record Level:** institutionCode: IZCAS-Ar45516
- **Type status:** Paratype. **Occurrence:** recordedBy: Taoqi Wang; individualCount: 1; sex: female; lifeStage: adult; occurrenceID: DDE0855A-075F-52B1-926E-CF494F9EEFF4; **Taxon:** scientificName: *Falcileptonetataoqii*; **Location:** country: China; stateProvince: Jilin; municipality: Tonghua; locality: Ji'an City, Dalu Town, Gaodi Village, Group 8, Feng Cave; verbatimElevation: 310.8 m; verbatimCoordinates: 125.8232°E, 47.0471°N; **Identification:** identifiedBy: Yejie Lin; dateIdentified: 2024; **Event:** year: 2024; month: 1; day: 24; habitat: Cave; **Record Level:** institutionCode: IZCAS-Ar45517
- **Type status:** Other material. **Occurrence:** recordedBy: Taoqi Wang; individualCount: 1; sex: female; lifeStage: adult; occurrenceID: 71F3DDEB-4035-5085-985C-3B9B3C2EE46C; **Taxon:** scientificName: *Falcileptonetataoqii*; **Location:** country: China; stateProvince: Jilin; municipality: Tonghua; locality: Ji'an City, Dalu Town, Gaodi Village, Group 8, Feng Cave; verbatimElevation: 310.8 m; verbatimCoordinates: 125.8232°E, 47.0471°N; **Identification:** identifiedBy: Yejie Lin; dateIdentified: 2024; **Event:** year: 2024; month: 12; day: 7; habitat: Cave

#### Description

**Male (holotype)**. Total length 2.16. Carapace 0.71 long, 0.86 wide. Eye sizes and interdistances: ALE 0.07, PME 0.06, PLE 0.05, ALE–PME 0.08, PLE–PME 0.03, PLE–PLE 0.09, AER 0.12, PER 0.15. Clypeus 0.16 high. Chelicerae with eight promarginal and four retromarginal teeth. Leg measurements: I 6.02 (1.66, 0.25, 1.85, 1.39, 0.87), II 4.48 (1.23, 0.22, 1.33, 0.95, 0.75), III (1.04, 0.21, -, -, -), IV 5.26 (1.46, 0.22, 1.61, 1.20, 0.77). Palp 1.34 (0.59, 0.19, 0.23, 0.33). Opisthosoma 1.20 long, 0.82 wide.

Colouration (Fig. 4A): Carapace yellowish-brown, without any pattern, cover with separated hair, edge darker; radial furrow obvious, lighter. Fovea longitudinal, reddish-brown. Chelicerae yellowish. Endites and labium yellowish-brown, bulging out medially, longer than wide. Sternum brown. Legs yellowish-brown without any pattern. Opisthosoma oval, yellowish-brown mottled with brown spots, dorsal lighter. Spinnerets yellowish-brown.

**Figure 2.**
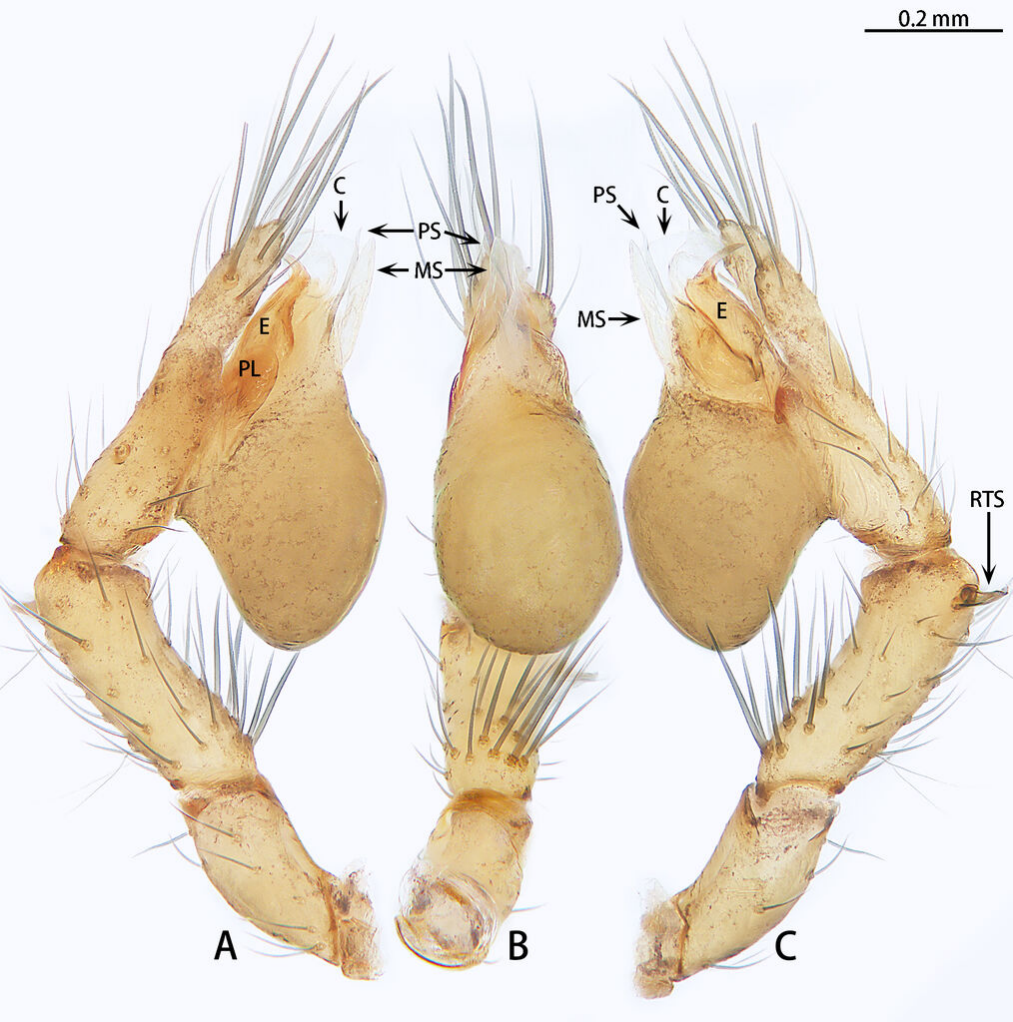
*Falcileptonetataoqii* sp. nov., holotype male. **A** Bulb, prolateral view; **B** Same, ventral view; **C** Same, retrolateral view. Abbreviations: C = conductor, E = embolus, MS = median sclerite, PL = prolateral lobe, PS = prolateral sclerite, RTS = retrolateral tibial spine.

Palp (Fig. 2A–C): Femur without row of strong spine, six times longer than wide. Patella slightly curved, three times longer than wide. Tibia base with 11 spines ventrally, with three trichobothria dorsally, one near cybium, two near patella, abreast. Two retrolateral tibial spines, dorsal spine shorter and darker than ventral spine, ventral spine semi-transparent, with a sharp and curved bending process apically. Tarsus unbranched, with a depression in the middle, apically blunt with dense of long setae. Bulb oval, almost 2.22 times longer than wide. Median sclerite transparent, blade-shaped, widest in the middle. Prolateral sclerite more sclerotised than median sclerite, straight laterally, but curved ventrally, needle-shaped. Conductor membranous, dorsal expanded in lateral view. Base of the embolus almost as wide as conductor, embolic tip strongly curved, sickle-shaped.

**Female (paratype).** Total length 2.23. Carapace 0.80 long, 0.69 wide. Eye sizes and interdistances: ALE 0.07, PME 0.06, PLE 0.06, ALE–PME 0.08, PLE–PME 0.03, PLE–PLE 0.08, AER 0.14, PER 0.15. Clypeus 0.15 high. Chelicerae with eight promarginal and five retromarginal teeth. Leg measurements: I 5.31 (1.46, 0.23, 1.61, 1.22, 0.79), II 4.08 (1.16, 0.24, 1.13, 0.88, 0.67), III 3.51 (0.96, 0.23, 0.91, 0.83, 0.58), IV 4.59 (1.36, 0.20, 1.44, 1.11, 0.48). Palp 1.42 (0.48, 0.14, 0.33, 0.47). Opisthosoma 1.30 long, 0.88 wide.

Colouration (Fig. 4B): Similar to that of male, except darker.

**Figure 3.**
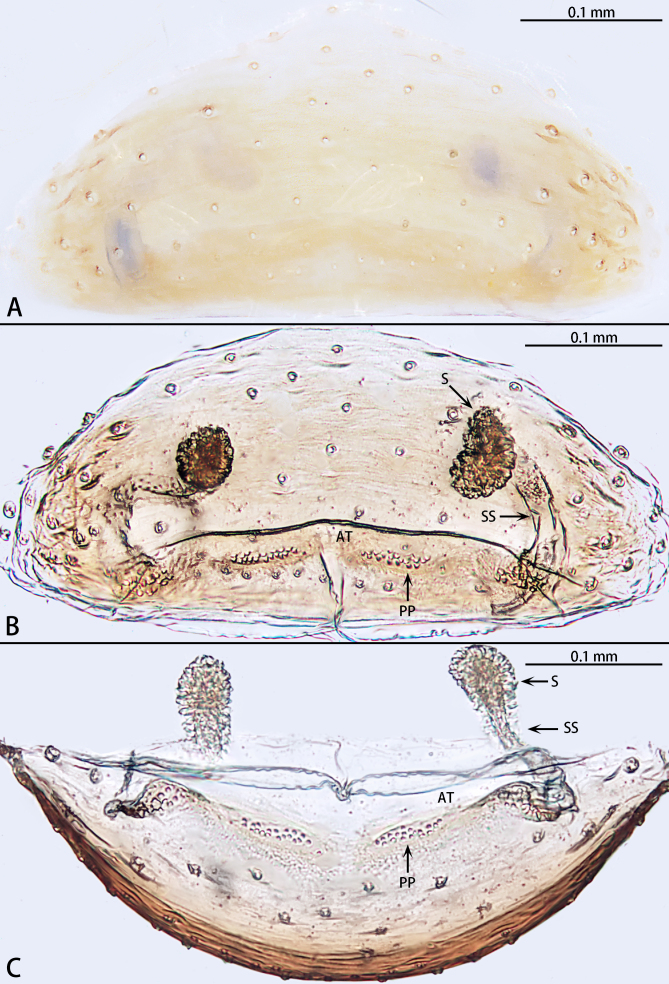
*Falcileptonetataoqii* sp. nov., one paratype female. **A** Epigyne, ventral view; **B** Vulva, dorsal view; **C** Same, posterior view. Abbreviations: At = atrium, PP = pore plate; S = spermathecae, SS = spermathecae stalk.

**Figure 4.**
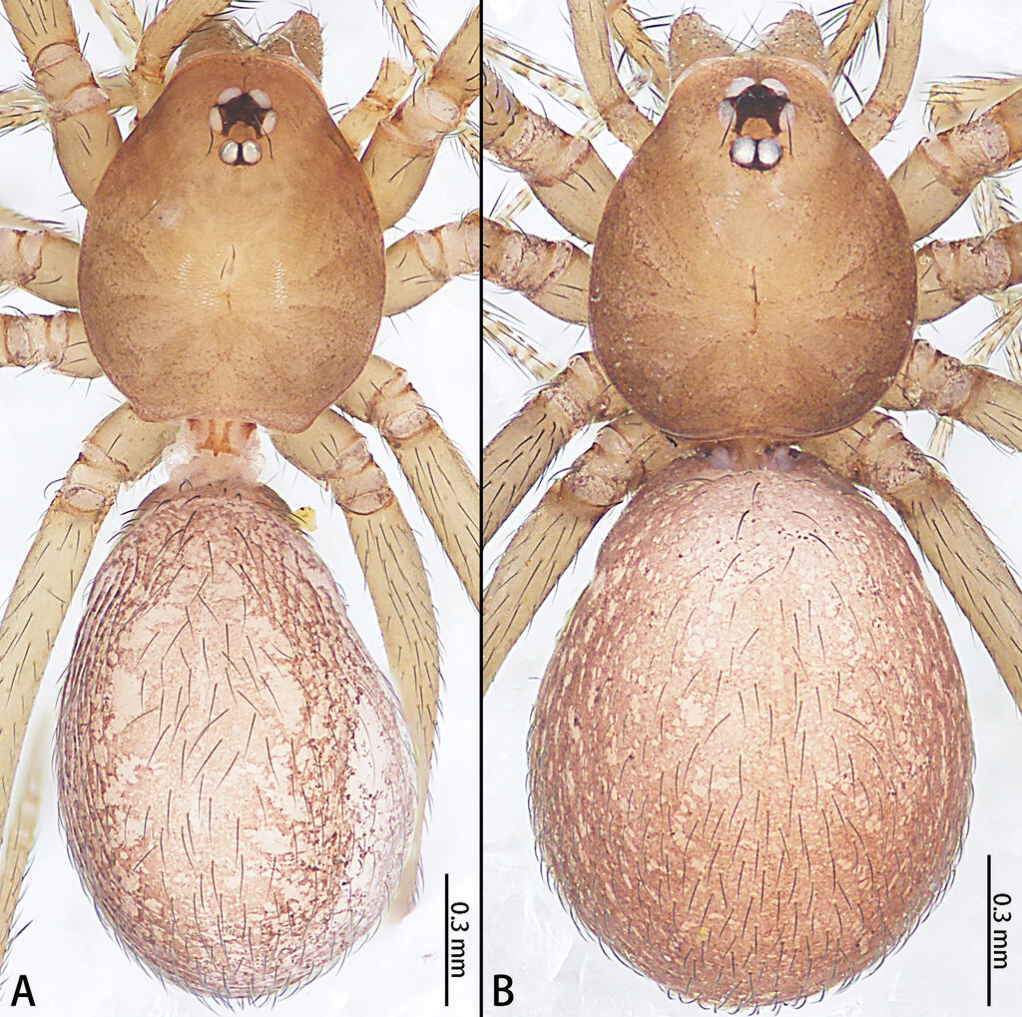
Habitus of *Falcileptonetataoqii* sp. nov., dorsal view. **A** holotype male; **B** paratype female.

Internal genitalia (Fig. 3A–C): Atrium rectangular, anterior margin of atrium flat. Pore plate oval, almost four times longer than wide. Spermathecae stalk without coiled, S-shaped. Spermathecae oval, heading dorsally, separated.

#### Diagnosis

Compared with the Chinese species, the male of *Falcileptonetataoqii* sp. nov. is similar to *F.lingqiensis* (Chen, Shen & Gao, 1984) by the tibia base with dense strong spines (Fig. 2A–C).

However, the males of the new species can be distinguished by the femur lacking a strong spine (Fig. 2A–C) [vs. present in *F.lingqiensis* (see Chen et al. (1984): figs. 7–9)] and tibia almost as long as cybium (Fig. 2A, C) [vs. shorter than the cybium in *F.lingqiensis* (see Chen et al. (1984): figs. 7 and 8)].

Compared with the South Korean species, the male of *F.taoqii* sp. nov. is similar to those of *F.bifurca* Seo, 2015, *F.cormuta* Seo, 2015, *F.innuta* Oh & Lee, 2023 and *F.sunchangensis* Seo, 2016 in two retrolateral tibial spines, prolateral sclerite sclerotised and needle-shaped and embolic tip narrowed and curved (Fig. 2A and C). Females of the new species are similar to those of *F.bifurca*, *F.innuta* and *F.sunchangensis* in the flat anterior atrium margin (Fig. 3).

However, male of the new species can be distinguished by the tibia base with dense strong spines (Fig. 2A–C) [vs. absent in *F.bifurca*, *F.cormuta*, *F.innuta* and *F.sunchangensis* (see Seo (2015): figs. 1B, 2H and F; Oh and Lee (2023): figs. 6C, D and F; Seo (2016): fig. 4H)], the tip of ventral retrolateral tibial spine curved (Fig. 2B and C) [vs. straight in *F.bifurca*, *F.cormuta* and *F.sunchangensis* (see Seo (2015): figs. 1B, 2H and F; Seo (2016): fig. 4H)], the median sclerite wide, almost 4.5 times longer than wide (Fig. 2B) [vs. thin, almost ten times longer than wide in *F.innuta* (see Oh and Lee (2023): fig. 6F)], the middle of the embolus unexpanded in retrolateral view (Fig. 2C) [vs. expanded in *F.bifurca* and *F.cormuta* (see Seo (2015): figs. 1B and 2F)] and the length ratio of the embolus base to the embolus tip is 2:1 in retrolateral view (Fig. 2C) [vs. 3:1 in *F.bifurca* and *F.cormuta*, 4:1 in *F.innuta* and 3:2 in *F.sunchangensis* (see Seo (2015): figs 1B and 2F; Seo (2016): fig. 4H; Oh and Lee (2023): fig. 6F)]. The female of new species can be distinguished by the pore plate almost four times longer than wide (Fig. 3B) [vs. twice in *F.innuta* (see Oh and Lee (2023): fig. 7C)], the spermathecae stalk uncoiled (Fig. 3B and C) [vs. coiled once in *F.bifurca* and *F.innuta* (see Seo (2015): fig. 1D; Oh and Lee (2023): fig. 7C)] and spermathecae separated (Fig. 3B and C) [vs. close to each other in *F.bifurca* and *F.sunchangensis* (see Seo (2015): fig. 1D; Seo (2016): fig. 4I)].

#### Etymology

The specific name is a patronym in honour of the collector Mr. Taoqi Wang, which is a noun (name) in the genitive case.

#### Distribution

**Figure 5.**
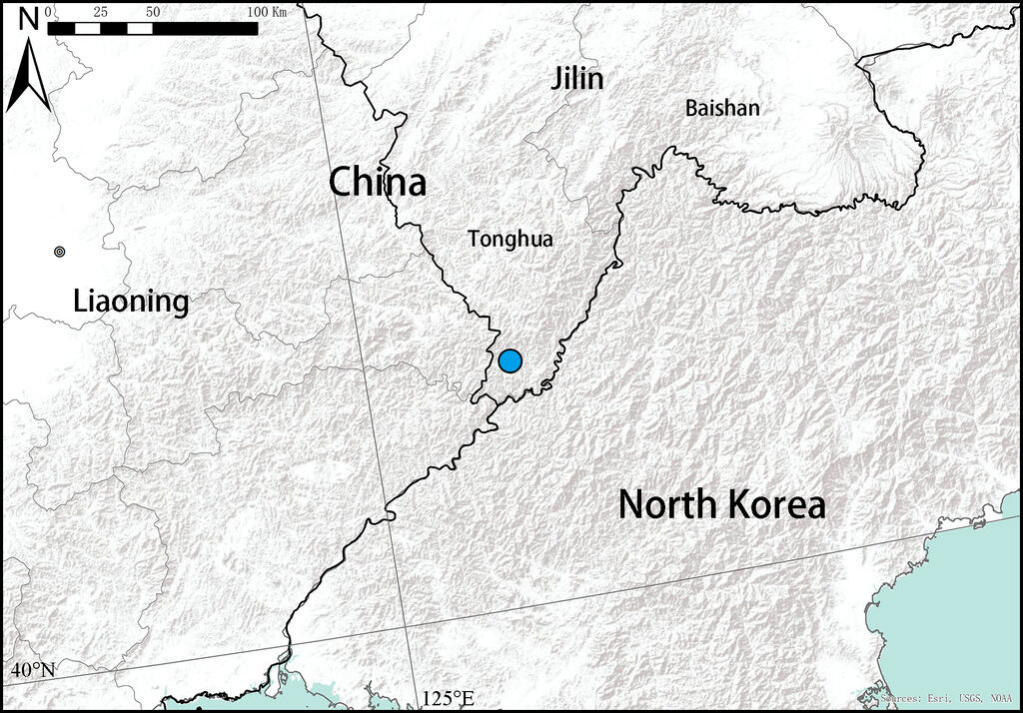
Distribution record of *Falcileptonetataoqii* sp. nov.

Known only from the type locality (Fig. 5).

#### Biology

All specimens were collected under stones in a cave (Fig. 1).

#### DNA barcode

ATAAGAGTAATTATTCGAATTGAATTAGGTCAGGGGGGTAGATTGATTGGAAATGATCATTTGTATAATGTAATTGTAACTGGACATGCTTTTGTTATAATTTTTTTTATGGTTATGCCAATTTTGATTGGGGGGTTTGGTAATTGATTGGTTCCTTTAATAGTGGGAGCACCTGATATAGCTTTTCCTCGCATGAATAATTTAAGATTTTGGTTGTTGCCTCCTTCCTTGTTTTTGTTGGTTATTTCTTGCATAGTGGAGATGGGTGCAGGTACTGGATGAACTGTATATCCTCCTTTAGCTTCTTATTTAGGTCATTCGTGATTGTCTGTAGATTTTGTTATTTTTTCTTTACATTTGGCAGGAGCTTCTTCTATTATGGGTGCTATTAATTTTATTACTACTATTGTGAATATGCGTGTTCATGGGATGTATATGGATAGGGTTACTTTATTTGTTTGGTCAGTATTAATTACTGCCGTTTTATTATTACTTTCTTTACCTGTATTGGCTGGAGCTATTACAATATTACTTTCTGATCGTAATTTTAACACTTCTTTTTTTGACCCTGCGGGGGGGGGGGATCCTATTCTATTTCAGCATTTGTTTT (GenBank accession number PQ777284).

## Supplementary Material

XML Treatment for Falcileptoneta taoqii
